# On the Reading, Recreation, and Amusements of the Insane
*Read before the American Association of Medical Officers of Hospitals for the Insane.


**Published:** 1853-10-01

**Authors:** John M. Galt

**Affiliations:** Physician to the Eastern Lunatic Asylum, Virginia, U.S. America


### ON THE READING, RECREATION, AND AMUSEMENTS OF THE INSANE.*

BY JOHN M. GALT, ESQ., M.D.,

*Physician to the Eastern Lunatic Asylum, Virginia, U.S. America.*

In accordance with my appointment at the last meeting of the Association, to report at the present session of this body, on "Reading, Recreation, and Amusements for the Insane," I beg leave to submit the following remarks.

Here, as on most points of treatment, we are led into great error, if we entirely abstract the insane from the sane, if we look upon the former class as altogether different in their psychological manifestations from the latter. Hence in making a just and proper basis, on which to originate alike our theoretical ideas and our practical operations, we must consider these two divisions of persons, though evidently differing in many particulars, as in the main holding the same position objectively with regard to reading, amusements, and recreation. Therefore it is, that if it be desirable to penetrate beneath the mere crust of the subject under consideration, it is requisite to take such a scope of inquiry as includes not only the insane, but also those who have the mind in an unimpaired condition.

The same general remark applies to reading as to two accompanying heads of our article; so far as regards the principle upon which it is employed as a moral means in the treatment of insanity, we adopt it as a measure which serves to occupy the mind to the effacement of delusions and morbid feelings, at least for a transitory period; it is, in other words, one of the great revulsive modes of acting upon the insane mind. Moreover, it serves as a pleasant method of passing away time, and in this respect exerts a tranquillizing effect on the individual. To many patients it thus proves a source of agreeable feelings, during time which would otherwise be full of the tedium of *ennui;* to the chronic insane, indeed, who have a taste in this direction, it is a source of comfort that beguiles many a lonely hour in the long and monotonous track of life's drear journey, spent away from the friends of their youth in the cloistered retirement of an asylum. In the third place, in some instances, besides the mere effect of reading as a portion of the treatment in mental alienation, instruction may also be acquired. It is, moreover, one of those means, the very provision of which, by the officers of an asylum, exhibits in a clear light the kindly disposition thoroughly felt by them towards their afflicted charge; thus having a tendency to lull all suspicious feelings on the part of the latter, at the same time that they are also rendered more manageable, by being contented and properly occupied.

Taking the sane mind as a point of departure in examining the utility and advantages of reading, we may proceed to divide the insane into several classes. In the first place, however, apart from the circumstance of insanity, we have some of these unfortunates who have never learned to read. These can be subject to this measure, then, only in two ways:—first, they may be taught to do so—a consideration not within the object of this report; and secondly, they may listen to the reading aloud of their fellow-patients and others. This latter method in a pauper asylum, and particularly in the institutions of States where education is not general, seems quite worthy of more attention than has been given it. Anecdotes in newspapers, and interesting tales may thus confer as much pleasure upon those incapable of reading, as upon those who possess this accomplishment.

* Read before the American Association of Medical Officers of Hospitals for the Insane.

With a number of the insane who have been educated, we take the broad ground of a nearly identical action from reading as occurs to the sane. For the diseased mind must not be considered as essentially morbid in all its faculties, but, as in numerous instances, having many of them, at least, in a comparatively undiseased condition. When, indeed, we look to the faint outlines which work the transition of sanity through what, in common language, is termed eccentricity, into positive and decided mental alienation, in other words, a state of insanity recognised as such not only by the expert, but by the most ordinary person, we cannot fail to arrive at the conclusion, that if reading is a source of pleasure and comfort to the sane and the eccentric, then may it be inferred, that it is so, too, to many of the insane. And what a comfort is found in this resource to a multitude of persons in all civilized countries; serving not only for instruction and religious counsel, but as a mode of abstraction from earth's cares and anxieties, as well as a pleasant recreation in the monotonous routine of daily life. The moments are few in which most of us can hang entranced on the stirring words that fall from the lips of genius, in conversation or from the oratorical tribune. But to the educated, by means of books, the wise and great, throughout all past time, and in all lands, speak as though face to face. Books, indeed, constitute a precious treasure-house of thought, which is open to all; which in every community has been a comfort to the sick and the afflicted; which has beguiled the watcher on the lonely sea, and the denizen of the crowded city; which has softened the hard lot of the prisoner, and added new charms to the life of the free in all its forms. Many of the insane, then, whose mental powers in this respect are not strikingly different from those of the sane, turn with eagerness to so potent a solace of weary hours. But with these, as with the sane, it should be remarked, there are some who, although they have received an education, are not fond of reading. The class of readers amongst the insane, who understand and appreciate what they read, nearly or quite as well as persons of sound mind, may be considered to pass gradually into another, who appreciate what they peruse but to a limited extent. Yet the effect of revulsive occupation is, perhaps, almost equally as great here as in the former class. With regard to such, an American superintendent observes—"For some, one book answers a long time. One day, recently, as I passed through the hall, an excited patient was lamenting that he had lost his sermon. The next day he was happy, he had found it; he said he had nearly read it through before my visit, and had only failed to do so the day before, when it was lost, for a long time previous. He said it was a good sermon, and he intended to read it through every day." A fourth division of the mentally deranged, which may be conventionally established, consists in that class of patients whose minds are so much weakened and affected, that they not only take no interest in reading, but appear not to comprehend or appreciate what they may still possess, the power of articulating in print. Reading may be considered, perhaps, as wholly inapplicable to these; and in the same division may be included lunatics whose minds are reduced to a state of idiocy, of greater or lesser completeness. To many patients, moreover, labouring under the acute or paroxysmal state of a maniacal attack, it is also manifestly inapplicable; considered merely as a *stimulus*, it likewise demands more caution in particular instances where there is much excitement.

As to conversation, change of scene, and other particulars, in connexion with the insane, so does the law laid down by Georget apply to reading. This is as follows:—"The ideas or passions of a patient should never be excited in the direction of his delusions." The application of the rule must be modified so as to suit each individual case. But, after all, the examples in which carefulness is necessary, cannot be considered as numerous. And on the whole (apart from a rule of supervision hereafter mentioned), I do not apprehend that there are many rules applying to the insane, with regard to reading, which differ essentially from those applicable to the sane. Thus all publications of an immoral tendency should be prohibited; and a pernicious influence is likely to ensue from too great a proportion of works of fiction. We think that, in general, the most suitable productions are such as combine interest, with no great strain upon the attention, or the reflective powers; for the insane are, many of them, incapable of persistent reflection for a long period, at least with pleasure; and, indeed, this is also the case with sane persons taken *en masse.*

We would not, by this remark, be understood as being against the adoption of schools in asylums, but the consideration of these does not enter into the field of our present inquiry. It should be observed, also, that where there is an acquaintance with any special art or science to a considerable extent, works of quite an abstruse character, and which to ordinary patients might prove very uninteresting, become in such instances just the contrary; and, indeed, the principle last enunciated is only modified here, inasmuch as where there is a familiarity with, and a love of, any subject, works relative thereto, however abstruse, may be considered to give pleasure, and at the same time, not as an unusual thing, to demand an excessive degree of attention. Hence such individuals should be furnished with productions of the character in question.

Every asylum ought to be provided with a library or collection of books. The principal portion of these should be, perhaps, travels, biography, history, and the many miscellaneous works which form the charming polite literature of the English tongue. In consonance with the character of the asylum, as to the number of patients therein who are acquainted with foreign languages, or who have been cultivators of the sciences, should be the amount of provision in this respect. And, indeed, in the library of every asylum, some few works of the kind are requisite. It being understood, also, that when a patient is received, as to whose peculiar pursuit, or department of study, there are no books in the library of such an institution, as great care should be exercised in the procuration of such as we would employ in purchasing an additional medicament to meet some rare physical symptom.

European writers concur with those of America as to the advantages of reading in asylums. The British commissioners in lunacy observe, for example, "No asylum should be without a library." And Dr. Jacobi remarks—"The collection of books belonging to the establishment must be of sufficient magnitude to satisfy the requirements of every case that occurs." These views we find, therefore, carried out to a considerable extent on the other side of the Atlantic, though not to so great an extent as in the United States. Nearly all the asylums in this country are furnished with a collection of books; and, as compared with foreign countries, a striking peculiarity in the reading matter is found in the large number of newspapers. Although British physicians not unfrequently mention in favourable terms the employment of newspapers and magazines, as, for example, in the reports of the asylums at Dundee and Hanwell,* I cannot omit this opportunity of adding my mite of admiration to the generous sympathy evinced by editors of the periodical press in this country, for the inmates of so many of our asylums; year after year this noble fraternity, though not often blessed with fortune's goods, continue to send their winged messengers to bear comfort to the hearts of the afflicted and despairing. The British commissioners complain of there being a deficiency of entertaining works in the asylums under their supervision, as compared with the abundance of religious productions, and they advise an alteration in this respect. The asylums in America vary in the number of books forming the library, from collections hardly entitled to such a name, to those institutions possessing several hundred volumes; and, in one instance, the number amounts

* The Saturday and Penny Magazines have been favourite publications in the British Asylums.

to more than a thousand. The staple of these collections consists, in general, of history, biography, travels, reviews, and such standard writers of fiction, as Sir Walter Scott, Miss Edgeworth, and Mrs. Sherwood. Dr. Thurnam observes, with regard to the York Retreat, in England,—"A reading-room, with a select library, consisting of books of travels, natural history, biography, history, and moral and religious works, has been provided for the men, the most orderly of whom have access to it under certain regulations as to conduct and behaviour. The books in this collection are also allowed to circulate among the women; and patients of more extensive acquirements and literary tastes have the opportunity of procuring the works of nearly all the standard authors from two excellent subscription libraries in the city." The last-mentioned idea has also been adopted elsewhere in Great Britain, and doubtless, when practicable, answers a good purpose in certain cases, particularly where we meet with some "*helluo librorum*," who will read through a comparatively small collection of books in a short time.

With regard to the perusal of the Bible by the insane, perhaps the following remarks, which we give from Dr. Chandler, respecting the patients in the institution of which he has charge, embodies the general views and practice of the physicians of our asylums:—"The Scriptures are placed in the hands of all whose disease does not lead them to make an improper use of them. Sometimes patients read and search the Bible to find passages to substantiate their delusions. Except in a few instances of this kind, the perusal of the Scriptures tends wholly to good, for therein is written the law of love and kindness, of justice and truth; and therein is taught nothing that vitiates the conscience, injures the health, or deranges the mind."

In some institutions a yearly fund is appropriated to the purchase of books, and this appears a good arrangement. The assistant physician is usually the officer who has charge of the library, and a certain day in the week is sometimes selected for giving out books. In many instances, however, not much system is observed in this respect, and the patients have pretty free access to the library throughout the week. The writer is decidedly in favour of a systematic action in this department, believing that analogous benefits are likely to result from such a course here, to those attending a similar procedure as to other things. Dr. Awl remarks respecting the Ohio Asylum,—"The rules which govern the library are amongst the best in our whole system, and we know of no more gratifying exhibition in the institution, than the orderly and interesting appearance of the different classes upon a sabbath morning, as parties from gallery after gallery arise and depart, with books of their own selection." The same physician elsewhere conveys the following significant hint to the friends of patients who send them newspapers:—"Avoid all papers that are filled with horrible suicides and murders. There certainly cannot be a greater mistake than to select articles of this character, and mark them with pencil, in order to attract their notice." At the Western Asylum of Virginia, in order to guard against impressions of this character systematically, it is the business of one of the officers to look carefully over the newspapers before they fall into the hands of the patients.

Although in most of our asylums a good deal of attention is given to the encouragement of reading, yet still perhaps we may not err in advising a systematic action in this respect—not contenting ourselves with merely providing the means of this pursuit to those who are anxious at all times to receive books and newspapers, but also in cases where there is torpor and apathy towards them, using due exertions to conquer this indifference. Moreover, there should be regulations tending to produce a proper degree of carefulness in the preservation of books; in other words, we should seek here that arrangement by which there is the maximum of reading, with as little attending wear and tear of material as possible, when conjoined with this paramount endeavour. A comfortable, pleasant reading-room, with some officer or attendant especially delegated to take charge of it, is, we think, a very desirable accompaniment of other facilities. This room should be furnished with books, newspapers, prints, illustrated works, maps, globes, &c.; and it might not be amiss to add also, such philosophical toys as the prism, the microscope, and the kaleidoscope. A rule which we cannot but view as highly important is, that the superintendent of an asylum should have a complete knowledge of the reading in which each of his patients engages. It is similarly important, that no part of the treatment here should escape his notice, as with regard to other means included under the head of moral management, there is sometimes a considerable degree of neglect in this respect. Perhaps also, with judicious oversight and selection, benefit might be derived from what is entitled a *course* of reading, as contra-distinguished from that of a desultory nature; this idea is rendered plausible, when we contrast the important influence of the one on the sane, when compared with that of the other.

A similar mode of reasoning applies to the basis upon which we originally adopt "recreation and amusements for the insane," to that whose existence we have endeavoured to demonstrate as sufficient ground for the employment of reading as a means of treatment. In other words, from the similitude of the diseased to the undiseased mind in many attributes, apart from all experience, we would be led *à priori* to the introduction of "recreation and amusements" in the treatment of the insane, from the facts connected with their habitual existence amongst sane persons. Looking through the long line of ages, even up to the primeval mystery which entailed labour thenceforth upon the race of man, we find parallel with constant industrial pursuits, the presence in every nation of modes of recreation and amusement. "Recreation," says Fuller, "is a second creation, when weariness hath almost annihilated one's spirit. It is the breathing of the soul, which otherwise would be stifled with continual business." The effect of a mistaken conscientiousness in endeavouring to interdict and abolish all means of recreation and amusement, only tends to disease of body and mind; and in order to restore the mental health of the insane, we discover by experience that the well-established necessity of such measures for the sound mind, is not found in vain as applied to the former unfortunate class. The general theory conventionally recognised as to the utility of amusements and recreation in the treatment of insanity, apart from the above considerations is, that by means of them we supplant the place of delusive ideas and feelings, tending by this disuse to their gradual enfeeblement or disappearance. The healthful influence of the hilarity attending such engagements, both upon the mind and upon the body, must also be allowed its due weight, and the general contentment ensuing from a continuous occupation of pleasant character. And so far as respects active amusements, the exercise involved, it is scarcely necessary to observe, must have a direct influence over the vital processes of the whole system. Moreover, for several reasons, there is a disposition in the insane to have their attention withdrawn to their own mental operations, rather than to enter into any intimate fellowship with each other. Amusements tend to break down this wall of separation, and by arousing social feelings, they wean the morbid spirit from so hurtful an introspection.

After Pinel and Tuke had substituted for the harshness of the old method of managing the insane, the two great measures, kindness and occupation, we soon find, in addition to bodily labour, the further recommendation by many writers, of recreation and amusements. These engagements had, it is true, been advised in hypochondriasis and melancholy by some of the older authors; but we can scarcely consider such counsel as of greater import than the loose suggestions and hints which, upon the occurrence of any valued discovery, are found, on examination, to gleam faintly from the misty chaos of the past, but which usually have little practical merit in heralding or assisting the actual and permanent establishment of a great improvement. Means acting upon the imagination, but serving too as recreation, had also been employed in ancient Egypt, at Gheel, and elsewhere in olden time; but the direct *intention* of these appears to have been simply to act as religious ceremonials.

All the standard writers on insanity may be cited in favour of amusements and recreation, as measures of treatment; and a variety is found in most of the asylums both of Europe and this continent. Those oftenest employed, are draughts, backgammon, cards, bagatelle, and chess. And with females, battledore, the graces, and swinging, are usual methods. In the American asylums, excursions in the neighbourhood of the institution by walking or riding, are amongst the most ordinary modes of recreation, especially in those of the south. This is not so much the case in Europe, public opinion being sometimes against it. Still occasionally the reverse occurs, as for instance, according to Dr. Browne, at the Montrose and Crichton Asylums in Scotland.

In looking over the writers on insanity, and the reports of asylums, we find that there is scarcely an amusement in which the sane commonly engage, that has not been recommended or adopted in some one or other asylum; and, in point of fact, the proper mode of reasoning as to the kind of amusement to be most advantageously followed, does not differ in the two classes, except as modified by circumstances almost extrinsic of insanity. Thus, in the first place, though there are some amusements not wrong in themselves, which may be engaged in by sane persons, and still not prove suitable, yet even here we will find conclusions apparently obvious, to be by no means without exceptions. For example, sailing by water, which at first view might seem too dangerous for the insane, is adopted at some asylums, and with few or no accidents ensuing from it. Dr. Anderson speaks of the use of a boat in fishing excursions, at the Haslar Naval Asylum, as being beyond all comparison the most valuable of remedial agents; and he states it his pleasing duty to report the complete success of a measure in the estimation of some fraught with so much danger to the lunatic by affording him an easy opportunity of carrying any suicidal propensity into effect. Dr. Kirkbride, it may be also mentioned, in the last report of the Pennsylvania Hospital for the Insane, alludes to excursions in steamboats by the patients of that institution. As regards the examples which might be given of this character, however, it would be better, so far at least as the idea applies to masses of the insane, and not to those treated in private, to be on the safe side, and rather avoid than otherwise modes of recreation attended with some risk.

In the second place, there are a few amusements which we may view as involving moral considerations, and which are employed as means of treatment in insanity. Cards we think unobjectionable as played by a large number of chronic cases; with these no ulterior bad effects can result, as they are destined most usually to pass their lives within the confines of an asylum. But in institutions where there are large numbers of young persons in the recent stage of the disease, perhaps some caution may be necessary lest a game be taught or encouraged, which might lead to gambling when they become sane. A second recreation, to which may be adduced some objections, is dancing. So far as regards persons of the same sex, we see no objections to this amusement; the mere active exercise in itself we believe will not be unsuitable, except in a few excited cases. The author of a work on dancing observes, that with most sorts of active amusement, the muscles of particular portions of the body are exercised and strengthened, but that it is somewhat at the expense of other parts, whilst in dancing the action is general and universally beneficial. Moreover, the prevalence of dancing amongst all nations evinces an inherent desire for grace in movement, and the pleasure attending its fulfilment. But it is a question whether it is not a bad mode of recreation, if the male and female patients dance in company, as having a tendency to arouse sexual feelings. There is a speculative idea respecting the character of the pleasure in dancing, which deserves notice here, and this is, that where the two sexes intermingle, a part of the pleasure seems to consist in a dim idea of the exclusive though temporary possession of the partner of the individual. We may remark that we are by no means in favour of an excessive rigour in preventing the mere sight of the opposite sex; we are satisfied, indeed, that the reverse of a good action will thus be created. By accustoming persons to see and meet the opposite sex continually, sexual feelings are rather lulled than the contrary; so that with the insane there is no objection, we think, to such occasional assemblages as will not be attended by any very direct intermingling, yet which at the same time accustom the sexes to the presence of each other. But so far as dancing is concerned, and perhaps anything in the nature of a mixed party, the intercourse between the two sections of patients becomes, we think, too free to prove advantageous. The experience and practice in different asylums, as to the amusement under consideration, is variable; in general, however, it is in some form rather approved of than the reverse.

Attendance on theatrical performances has been recommended by some writers. And plays have been acted by the insane in a few instances, both on the continent of Europe, and in the United States. On this subject opinion seems divided. One caution is evidently requisite here, that such scenes should be avoided as tend to excite into action the sexual feelings. The effects on the insane performer would seem to be chiefly those of mental revulsion. It is also, however, a question of wide import, how far will prove beneficial or otherwise, the gratification of the love of display, the awakening of emulative feelings, the fear, the hope, and other emotions attendant upon the exercises of the rostrum and the stage. The recreative effect on the insane as spectators must be in general simply the action of other public exhibitions, except that in the case of tragedy, or at a public theatre, the excitement of the emotions might be great. Farce and comedy would seem to be the most suitable performances for such actors and spectators. The experience of Esquirol was decidedly against the institution of theatrical performances, this being the conclusion which he drew from the effects on the insane at Charenton, attendant on the exhibitions given by them for several years. Dr. Brown, on the other hand, so far as regards the consideration of this class of persons merely as spectators, observes, respecting the patients of the Crichton Asylum in Scotland, that the theatre has been an object of great attraction to them, and that this mode of recreation was encouraged for many reasons; but chiefly because the drama conveys much amusement, and some information, without imposing either sustained mental exertion or attention, supplying pastime without passion, and knowledge without study, suggesting truth by fiction, and appealing to the happy, the cheerful, and the mirthful parts of our nature.

Amidst the numerous amusements which have been recommended or employed with the insane, it would be well, we think, to select those which refer themselves rather to grown persons than those which are suitable for mere children.

Means falling rather under the head of labour than amusement, answer very well to recreate the minds of some patients, who have a taste for such pursuits. Thus the mental occupation accompanying study acts in this way; gardening is also of this character; and with females, ornamental needlework. A taste for the fine arts should be fostered by providing the necessary means of pursuing them. In a description of the asylum at Palermo, in Sicily, a medical acquaintance of mine observes, that this establishment is ornamented with paintings and statues, the work of the insane residents.

Music, we think, deserves every encouragement, and, indeed, it constitutes a very common pastime in most of our asylums; whiling away the hours agreeably in individual instances, and serving to enliven routine assemblages of patients, or being employed as a preparation for the sabbath exercises of the choir. The directly curative effects of music, of which the older writers have spoken, do not appear to be borne out by experience. It may be doubted, however, whether a sufficiently *scientific* and well-arranged trial of this means has yet been made, in the first place, as to its direct action on the nervous system, and secondly, merely as a mode of occupation. Some of the older writers make particular mention of the kinds of instruments and airs which are most suitable in each variety of insanity, and they enter into considerable details on the subject. This seems, however, to have been of little consequence. Respecting the direct action of music on the nervous system, Esquirol says, "a small number of instruments should be selected, the musician should not be seen by the patient, and airs familiar to his infancy, which were agreeable to him before his disease, should be executed." As being ready and pleasant means of obtaining musical sounds, we would recommend the employment in an asylum, of a musical-box, hand-organ, and æolian harp. For singing in large numbers together, perhaps temperance songs would answer a good purpose; inasmuch as with some persons there exists a religious dislike of any such *vanities* as ordinary songs. Most certainly no exertions made by the officers of an asylum can be deemed as in vain, which have for their end the cultivation of the mental faculty, on which this art depends, in any patient where the least success is to be hoped. We would recommend, therefore, greater efforts in this particular. It may be observed, also, that the Mainzerian system of singing, as adopted in several British and continental asylums, deserves being employed in those of the United States.

About every institution for the insane there should be as many objects as possible to interest and excite the attention. Thus the grounds should be extensive, and adorned with flowers; a fish-pond and playing fountain are not amiss here in the way of ornament. There should also be woods with agreeable walks in them, and fitted up with summer-houses and convenient seats and swings; a deer-park is sometimes found; and we may have, too, a cottage furnished with books, maps, and curiosities. A museum is also an interesting addition to an asylum, particularly if designed chiefly for the educated classes. Of a similar character is a greenhouse. In some of the British asylums, for the sake of the surrounding scenery, there is a mound placed in the centre of the court. Terraces are also not unfrequently found. Walking and riding out to attractive spots, and similar modes of recreation, it may be observed, are common in American asylums. Different exhibitions occurring in the vicinity of the asylum, have, too, in some instances, been visited by the patients.

A requisite part of every good asylum is at least one very large room of a square, or other convenient form; by reason of the fact that assemblages of the patients for different purposes now constitutes an engagement common in most of our asylums. Thus lectures are given in a few instances, and, it is reported, with very beneficial effects. Again, there are exhibitions with the magic lantern, dissolving views, and other modes of amusing large numbers at a time. Or such an apartment will prove useful for the recitations and other exercises connected with those asylums in which schools form a means of treatment; or for the debating societies that have sometimes been adopted with advantage; and also for musical parties, and other social re-unions. Both in Great Britain and this country, moreover, it is sometimes a custom to celebrate the public anniversaries after the manner of the sane; and the associations and cheerfulness thus aroused, have often been alluded to with commendation; in this regard a large room is quite useful. Apart from the mere amusement in all these various gatherings, it may be observed, that an important end is attained with the insane by increasing their self-control, and their power over the will. I cannot but approve here of the arrangement adopted in some asylums, of allotting to each day of the week some particular mode of recreation or mental employment, thus preserving a continuous pleasing influence, and keeping up an unbroken revulsive action, whilst, besides other good effects, the evils ensuing from *ennui* are entirely removed.

In addition to the modes of amusement previously mentioned, it may not be amiss to run over a list of most of the remainder, which have been adopted in different asylums. These are dominoes, the mansion of happiness, Dr. Busby, the Pickwick cards, fox and geese, German tactics, monis, the game of the mill, dissected maps, Chinese puzzles, summer ice, billiards, bowls, nine-pins, and its modification one pin, the ring, marbles, coronella cups, tennis, different games played with a ball, the see-saw, the spring-board, the rocking-boat, the rocking-horse, the jumping-rope, the ginstra, flying-horses, the miniature rail-road, and quoits. Fishing and similar excursions into the surrounding country are also not unfrequently allowed; and even bathing in summer. Training animals has been likewise deemed beneficial. In Great Britain, cricket and foot-ball, amusements common with the sane, have been adopted at some of the asylums. In private practice travelling is occasionally of benefit, and this has been recommended by many writers in cases of melancholy and hypochondriasis; but there is not entire agreement on this head, amongst those well acquainted with the phenomena of mental disease. There are other modes of passing time agreeably, which require no other provision for carrying them on, or but very little, except the inclination of the parties concerned; some of these will also be found suitable to the insane; narrating stories to them, for example, has been occasionally employed with advantage. Such diversions as, "What are my thoughts like?" "How do you like it?" and the game of "definitions," might, too, be made to serve as recreative means with some.

Proportionately as asylums differ in the number of their patients, the character of the inmates, as to original disposition and tastes, and other circumstances, so will there be greater or less advantage from the introduction of one or other particular kind of amusement. Difference of management and national characteristics here exert an important bearing; thus an amusement which has failed in the asylum of one nation, might succeed in that of another; nay more, that which had proved useless in one gallery of an institution, might be decidedly beneficial in another, with different patients and different attendants. It well behoves a superintendent to study the relative position, in this aspect, of the institution of which he has the supervision, and to adopt a corresponding action. The number of recent cases, for example, is an important item, connected as it is, when extensive, with a large body of convalescent patients. We may indeed suppose, in this instance, a new element added to any of our calculations, consisting not so much of insane persons as of the sane—who may be expected to aid us in contriving and carrying into effect various modes of recreation designed for our inmates generally.

Whatever be the character of the institution, however, a second indication which the superintendent should have in view, is to be ever on the watch to stimulate and encourage any innocent mode of recreation which patients may themselves devise, or which may be suggested by others; and he should carefully seek to try every plausible means of adding to the comfort and welfare of his afflicted charge in the way of recreation—also directing his attention to borrow hints on the subject from the pursuits of the sane.

But, moreover, we should carefully avoid the error of using little effort, except merely providing the means of amusement. Many of the insane are indifferent as to any diversion, and indeed to all other kinds of exertion, either mental or bodily. Hence we should use every mode of encouragement, both by example and persuasion, and by furnishing a variety of diversions in order to satisfy the taste of each one. As is the case with sane persons, we may anticipate that the insane will become tired of particular kinds of amusement, and hence we should always have this in view, so as to supply the place of one by that of another. In fine, the most systematic efforts should be continually made, by furnishing adequate means of amusement and recreation, to prevent the insane from lapsing into the dull torpor of reverie and indolence, into which it is the very nature of man to sink, if mind and body alike are left in a state of vacuity, from the want of means to occupy them.

